# Genetic microevolution of clinical *Candida auris* with reduced Amphotericin B sensitivity in China

**DOI:** 10.1080/22221751.2024.2398596

**Published:** 2024-09-05

**Authors:** Sufei Tian, Chen Rong, Hailong Li, Yusheng Wu, Na Wu, Yunzhuo Chu, Ning Jiang, Jingping Zhang, Hong Shang

**Affiliations:** a National Clinical Research Center for Laboratory Medicine, State Key Laboratory for Diagnosis and Treatment of Infectious Diseases, Department of Laboratory Medicine, the First Hospital of China Medical University, Shenyang, People’s Republic of China; b NHC Key Laboratory of AIDS Prevention and Treatment, The First Hospital of China Medical University, China Medical University, Shenyang, People’s Republic of China; c Department of Infectious Diseases, the First Hospital of China Medical University, Shenyang, People’s Republic of China

**Keywords:** *Candida auris*, reduced AmB sensitivity, RNA-Seq, Bladder irrigation, ERG

## Abstract

The global rate of Amphotericin B (AmB) resistance in *Candida auris* has surpassed 12%. However, there is limited data on available clinical treatments and microevolutionary analyses concerning reduced AmB sensitivity. In this study, we collected 18 *C. auris* isolates from five patients between 2019 and 2022. We employed clinical data mining, genomic, and transcriptomic analyses to identify genetic evolutionary features linked to reduced AmB sensitivity in these isolates during clinical treatment. We identified six isolates with a minimum inhibitory concentration (MIC) of AmB below 0.5 µg/mL (AmB^0.5^) and 12 isolates with an AmB-MIC of 1 µg/mL (AmB^1^) or ≥ 2 µg/mL (AmB^2^). All five patients received 24-hour AmB (5 mg/L) bladder irrigation treatment. Evolutionary analyses revealed an *ERG3* (c923t) mutation in AmB^1^ *C. auris*. Additionally, AmB^2^ *C. auris* was found to contain a t2831c mutation in the *RAD2* gene. In the AmB^1^ group, membrane lipid-related gene expression (*ERG1, ERG2, ERG13,* and *ERG24*) was upregulated, while in the AmB^2^ group, expression of DNA-related genes (e.g. *DNA2* and *PRI1*) was up-regulated. In a series of *C.auris* strains with reduced susceptibility to AmB, five key genes were identified: two upregulated (*IFF9* and *PGA6)* and three downregulated (*HGT7, HGT13,*and *PRI32)*. In this study, we demonstrate the microevolution of reduced AmB sensitivity in vivo and further elucidate the relationship between reduced AmB sensitivity and low-concentration AmB bladder irrigation. These findings offer new insights into potential antifungal drug targets and clinical markers for the “super fungus”, *C. auris*.

## Introduction

*Candida auris* is rapidly emerging as a global pathogen due to its increasing incidence of drug resistance. Amphotericin B (AmB) is typically employed as the last line of defense in treating fungal infections, particularly in patients with *C. auris* infections that are resistant to echinocalcins or unresponsive to echinocalcins [1]. Unlike common Candida species or other yeasts, resistance to AmB, including in haploid organisms such as *Candida glabrata* remains extremely rare despite over 50 years of clinical use [2,3]. Recent meta-analyses have revealed an AmB resistance rate of 12% in *C. auris* isolates [4]. Currently, the molecular basis underlying reduced susceptibility to AmB or AmB resistance in any yeast species is poorly understood. Studies have indicated that the molecular mechanisms by which *Candida* spp. develops resistance to AmB are related to specific target genes, mainly *ERG2, ERG3, ERG6,* and *ERG11* [5–11]. One study identified a mutation in *ERG6* as a cause of AmB resistance in clinical strains of *C. auris* [12]. Additionally, transcriptional profiling of AmB-resistant isolates has shown a significant difference in the expression of ergosterol biosynthesis genes, suggesting that AmB resistance may be associated with changes in membrane lipid permeability and chromatin remodelling [13]. The aforementioned studies focused exclusively on AmB-resistant strains. Therefore, in this study we analyzed strains with elevated minimum inhibitory concentrations (MICs) for AmB as well as a series of strains at different evolutionary stages (i.e. AmB MIC ≤ 0.5μg/mL, AmB MIC = 1μg/mL, and AmB ≥ 2 μg/mL) to comprehensively investigate the microevolutionary mechanisms behind reduced AmB sensitivity.

We observed that these strains appear to be associated with the clinical application of AmB therapy (i.e. intravenous administration, atomized inhalation, and bladder irrigation), warranting further investigation. However, in China, there have been limited studies on AmB resistance [14,15]. Continuous surveillance in recent years has revealed the emergence of several strains of AmB-resistant *C. auris* in Shenyang, China. Given that studies have shown high levels of AmB resistance in *C. auris* following AmB intravenous administration [12], we initially focused on investigating cases of *C. auris* strains with reduced sensitivity as indicated by AmB MICs.

In this study, we systematically investigated the clinical characteristics of a series of AmB-resistant strains, as well as the evolutionary features of these strains, using genomic and transcriptomic analyses to uncover the evolutionary basis of AmB resistance in clinical *C. auris*.

## Methods

### Isolates

Clinical isolates of *C. auris* were regularly isolated from Sabouraud dextrose agar or CHROMagar *Candida* medium and incubated at 37 °C under atmospheric conditions from January 2019 to September 2022. Antifungal susceptibility testing of all clinical *C. auris* isolates was performed by broth microdilution (ATB Fungus 3, BioMerieux France). The MIC endpoints for AmB were defined as the lowest drug concentration that caused 100% growth inhibition after 24 h of incubation at 35 °C. Clinical *C. auris* isolates were screened for drug sensitivity, and those with MIC values for AmB greater than 0.5μg/mL were included in the analysis. Further, we also included AmB-sensitive strains originating from the same patient for comparative analysis. We performed the skin screening (axilla and groin) on September 15th, 2022 in the two cases relevant to this study (Supplementary Table 1).

In addition, all strains of this experiment were tested for sensitivity to drugs such as echinocandins by applying a commercial chromogenic susceptibility plate (Sensititre YeastOne, Thermo Fisher Scientific). *Candida parapsilosis* ATCC22019 and *Candida krusei* ATCC6258 were used for strict quality control during testing.

### Case patient information

Patient information data, including sex, age, ward, underlying diseases, and antifungal drugs administration (including AmB intravenous, atomized inhalation, and bladder irrigation), and antifungal drug dosage, were collected via the Hospital Information System of the First Hospital of China Medical University. This study was approved by the first hospital of China Medical University (ERC No. 2024-41). Clinical samples were obtained after verbal consent only as part of routine patient care and diagnostic workup for pathogen isolation and susceptibility testing. The case diagram was generated on https://app.diagrams.net.

### Genome sequencing

Genome sequencing was performed by Shanghai Personal Biotechnology Co., Ltd using the Illumina NovaSeq platform as described previously [14]. For variant calling analysis, the reference genome (*C. auris* Respiratory Intensive Care Unit (RICU)1_A1) was downloaded from the National Center for Biotechnology Information (NCBI) genome database (NCBI accession: ASM1421745v1). The variants that were predicted to alter the amino-acid sequence in any coding sequence (nonsynonymous single nucleotide variants, stop loss, or gain variants, as well as indels) were annotated using the ANNOVAR software [16] and the RefSeq *C. auris* B11221 coding sequence.

### Phylogenetic analysis

The maximum likelihood method based on the Tamura-Nei model was used to predict evolutionary history [17]. The location of blank and missing data was cleared for analysis and MEGA7 [18] software was used for the evolutionary analyses. A phylogenetic tree was constructed using https://www.chiplot.online/normalTree.html. Protein–protein interaction (PPI) network analysis of non-mutated genes was conducted using the STRING database (https://cn.string-db.org). PPI network diagrams were constructed on https://www.chiplot.online/normalTree.html.

### Transcriptome analyses

RNA sequencing analyses were performed using the Illumina NovaSeq platform (Shanghai Personal Biotechnology Co.,Shanghai, China). For RNA extraction, *C. auris* cells were grown in YPD broth medium at 30 °C in a shaking incubator at 220 rpm. After 18 h, cells at the stationary phase were diluted with an equal volume of fresh YPD broth and incubated for 2 h at 37 °C to induce growth. Cells were then centrifuged for 10 min at 3000 × g before pellets were flash frozen and stored at −80°C. Total RNA was purified using a GeneJET RNA purification kit (Thermo Scientific) before RNA quality was assessed on a Bioanalyzer using the RNA6000 Nanochip (Agilent). Next, mRNA was enriched using oligo (dT) beads (New England BioLabs (NEB). Subsequently, double-stranded cDNA libraries were generated using the NEBNext Ultra directional RNA library prep kit for Illumina (NEB) according to the manufacturer’s instructions. The qualified libraries were subjected to Illumina sequencing with 150 bp paired end reads at the Novogene sequencing facility. Three biological replicates were included for each condition.

For data mapping analysis, the reference genome (*C. auris* B11221) and gene annotation files were downloaded from genome website. Differentially expressed genes (DEGs) were screened for expression difference fold |log_2_FoldChange| > 1, and a significant adjusted *p-value *< 0.05.

Several important genes were selected for qPCR validation and the primers are shown in Supplementary 3.

## Results

### Clinical isolates and patient information

A total of 18 *C. auris* isolates were obtained between January 2019 and September 2022, including 13 from urine specimens, one from cerebrospinal fluid, and four from the skin. All the isolated strains exhibited high levels of resistance to fluconazole and sensitivity to 5-fluorocytosine. The MIC ranges for voriconazole and itraconazole were 1–4 μg/mL and 0.125-0.5μg/mL, respectively (Supplementary Table 1). Given that the MIC values for the two QC strains (*Candida parapsilosis* ATCC22019 and *Candida krusei* ATCC6258) tested by Sensititre YeastOne (SYO) against AmB were 1–2 dilutions higher compared to those tested by Fungus3 (F3), and considering that other studies suggest SYO overestimates AmB resistance in *C. auris* [19], we used MIC values from the F3 test for subsequent experiments. *C. auris* strains were grouped according to their MIC values for AmB as follows: ≤ 0.5, 1 μg/mL, and ≥ 2 μg/mL, designated as AmB^0.5^, AmB^1^, and AmB^2^, respectively. Strains in the AmB^0.5^ group included RICU40_A425, RICU41_A431, RICU43_A478, RICU40_Y024, RICU37_S006, and RICU37_S010. The AmB^1^ group included RCIU38_A382, RICU40_A441, RICU40_A447, RICU40_A451, RICU41_A454, and RICU41_A457. The AmB^2^ group consisted of six strains: Neurosurgical Intensive Care Unit (NSICU) 2_A120, RICU38_A397, RICU40_Y021, RICU43_A485, and RICU43_A485, which had an MIC of 2 μg/mL for AmB in the F3 assay and 4 μg/mL in the SYO assay. The remaining strain, RICU38_A398, had MICs of 4 μg/mL in the F3 assay and 8 μg/mL in the SYO assay. According to the Centers for Disease Control's tentative breakpoint, *C. auris* is considered AmB-resistant if the MIC is higher than 2 μg/mL. Therefore, the six strains in the AmB^2^ group should be regarded as AmB-resistant.

**Figure 1.**
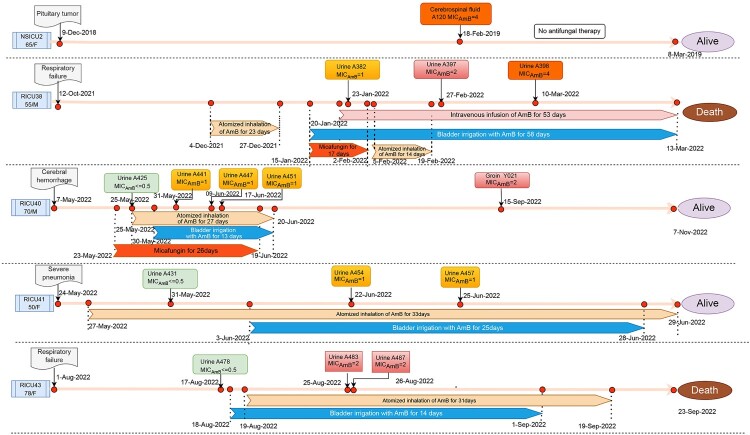
Treatment regimens and isolates collection for five cases: one without AmB treated and four AmB treated patients. AmB, Amphotericin B; MCF, Micafungin. The rectangles above the timeline represent isolated *C. auris* with different MICs. Green rectangle: AmB ^0.5^ isolates, yellow rectangle: AmB ^1^ isolates, pink and orange rectangle: AmB ^2^ isolates. The lower part of the timeline represents the different treatments. Blue arrow: AmB bladder irrigation, brown arrow: AmB atomized inhalation, pink arrow: AmB intravenous drip.

Based on these strains, we retrospectively reviewed relevant cases (Figure 1). Only one strain was isolated from a NSICU patient, while the remaining isolates were all obtained from RICU patients*.* With the exception of Case 1, in which the patient was not treated with any antifungal drugs for cerebrospinal fluid *C. auris* infections, all four patients were empirically initiated on 5 mg/L/12 h AmB atomized inhalation for respiratory tract *Candida* infections, followed by 5 mg/L/24 h AmB continuous bladder irrigation. Reduced AmB sensitivity gradually emerged following AmB bladder irrigation, while all respiratory cultures remained negative for *C. auris* after a course of respiratory atomized inhalation. During intravenous treatment with AmB, five blood cultures were taken from RICU38 and sent for testing, all of which were negative.

### Case 1

NSICU2, a 65-year-old female, was admitted to the neurosurgery department on December 9th, 2018, due to a pituitary tumour. *C. auris* was cultured from her cerebrospinal fluid for the first time on February 18th, 2019. The isolate, A120, had an MIC of 4 μg/mL for AmB, indicating drug resistance. No antifungal drugs were used and cerebrospinal fluid specimens were not examined again. The disease did not worsen 18 days after the isolation of *C. auris* (A120), which was considered colonization rather than infection. The patient was discharged from the hospital on March 8th, 2019.

### Case 2

RICU38, a 56-year-old male, previously diagnosed with hypertension and diabetes, was admitted to the ICU of the Department of Respiratory and Critical Care Medicine on October 12th, 2021, due to a right thalamic hemorrhage, Guillen Barré syndrome, and repeated pulmonary infections. During hospitalization, the patient received ventilator-assisted ventilation and anti-infection treatment. From October to December 2021, *C. albicans* and *C. glabrata* were repeatedly cultured from sputum samples. Fluconazole (0.4 g/24 h) was administered intravenously, and AmB (10 mg/12 h) was given as atomized inhalation therapy. No *Candida* growth was observed in the sputum after treatment. On January 13th, 2022, *C. auris* was isolated and cultured from the patient’s urine samples for the first time (AmB MIC ≤ 0.5 μg/mL), suggesting an AmB-sensitive *C. auris* urinary infection. On January 15th, 2022, he was given Micafungin 150 mg for 17 days (from January 15th, 2022, to February 2nd, 2022) due to Candida lung infection. On the same day, the patient was then treated with continuous bladder irrigation with AmB (5 mg/L) for 24 hours. On January 23rd, 2022, *C. auris* A382 (AmB MIC = 1 μg/mL and resistant to echinocandins) was isolated from the patient’s urine culture. Continuous bladder irrigation with AmB was administered until March 13th, 2022, followed by 5 mg of intravenous AmB infusion. *C. auris* A397 (AmB MIC = 2 μg/mL) and A398 (AmB MIC = 4 μg/mL) were isolated from urine specimens on February 27th and March 10th, respectively. Reduced AmB sensitivity gradually increased during irrigation, with MIC values rising from 1 μg/mL to 4 μg/mL. Finally, due to septic shock and multiple organ dysfunction, the family stopped further treatment and the patient left the hospital on March 13th, 2022.

### Case 3

RICU40, a 70-year-old male patient, underwent right fossa dural arteriovenous fistula amputation and right temporo-parietal hematoma removal on April 19th, 2022. This patient also suffered from repeated postoperative pulmonary infections. He was admitted to the RICU on May 7th, 2022, and was placed on tracheal intubation with ventilator-assisted ventilation during hospitalization. On May 12th, the patient developed a fever and elevated inflammatory markers such as CRP and PCT. Anti-inflammatory therapy was administered with cefoperazone-sulbactam (3 g/8 h) and meropenem (1 g/6 h), but symptoms did not improve. In May 2022, repeated sputum cultures indicated *C. auris* growth (AmB MIC ≤ 0.5 μg/mL), and AmB (5 mg/12 h) atomized inhalation therapy was initiated. Micafungin 150 mg (2022-5-23–2022-6-19) was given to treat the *C. auris* lung infection for 26 days. *C. auris* A425 (AmB MIC ≤ 0.5 μg/mL) was isolated and cultured from urine samples for the first time on May 25th, 2022, and continuously isolated in urine thereafter. On May 30th, 2022, 24-hour AmB (5 mg/L) continuous bladder irrigation was added to the patient’s treatment regimen. Subsequently, *C. auris* A441 (AmB MIC = 1 μg/mL), A447 (AmB MIC = 1 μg/mL), and A451 (AmB MIC = 1 μg/mL), all with decreased AmB sensitivity, were continuously isolated from urine samples. During the screening of the patient's skin on September 15th, 2022, *C. auris* Y021 (AmB MIC = 2 μg/mL) was isolated from the patient's groin, and axillary strain Y024 (AmB MIC ≤ 0.5 μg/mL) was isolated. At the same time, S010 and S006 were isolated from the groin and axilla of a neighbouring RICU5 patient. After a period of treatment, the patient was discharged from the hospital on November 7th, 2022.

### Case 4

RICU41, a 49-year-old female patient, underwent right meningioma resection in the neurosurgery department in March 2022 and suffered from repeated postoperative pulmonary infections. She was admitted to the RICU on May 24th, 2022 and was placed on tracheal intubation with ventilator-assisted ventilation during hospitalization. In May 2022, *C. glabrata* was repeatedly isolated from sputum specimens, so the patient was given atomized inhalation therapy with AmB (5 mg/12 h) on May 27th. *C. auris* A431 (AmB MIC ≤ 0.5 μg/mL) was isolated from urine samples for the first time on May 31st. On June 3rd, the patient’s catheter was replaced, and 24-hour AmB (5 mg/L) continuous bladder irrigation was administered. Following anti-infection treatment, the patient’s infection index and fever symptoms were improved, but the urinary tract infection persisted, and the indwelling urinary tube was removed on June 20th, 2022. *C. auris* A454 (AmB MIC = 1 μg/mL) and A457 (AmB MIC = 1 μg/mL) were isolated from urine samples on June 22nd and June 25th, 2022. After a period of treatment, the patient's fever symptoms and liver function improved significantly, and she was discharged on June 29th, 2022.

### Case 5

RICU43 was a 78-year-old female with diabetes mellitus and hypertension. Intermittent dyspnea had occurred for nine months before admission, and no complete improvement was observed after anti-infection and ventilator-assisted ventilation treatment. Intravenous therapy was administered with 50 mg tigecycline, 0.5 g/q.d. levofloxacin, and 1.0 g/q.8 h meropenem. The patient was transferred to the RICU on August 1st, 2022, due to respiratory failure and severe pneumonia. During hospitalization, she was placed on tracheal intubation with ventilator-assisted ventilation. *C. albicans* was repeatedly cultured from sputum samples in the early stage of admission, and fluconazole (0.4 g/24 h) antifungal therapy was administered. From August 15th to September 22nd, *C. auris* (AmB MIC ≤ 0.5 μg/mL) was continuously isolated from the patient's sputum, and because these isolates showed sensitivity to AmB, the patient was treated with 5 mg/12 h AmB as an atomized inhalation therapy. *C. auris* A478 (AmB MIC ≤ 0.5 μg/mL) was isolated and cultured from urine samples for the first time on August 17th, and the patient was treated with 24-hour AmB (5 mg/L) continuous bladder irrigation. On August 25th and 26th, *C. auris* A483 (AmB MIC = 2 μg/mL) and A485 (AmB MIC = 2 μg/mL) were isolated. After this, the patient’s condition became critical due to septic shock and multiple organ failure. Subsequently, the family declined further treatment, and the patient left the hospital on September 23rd, 2022.

**Figure 2.**
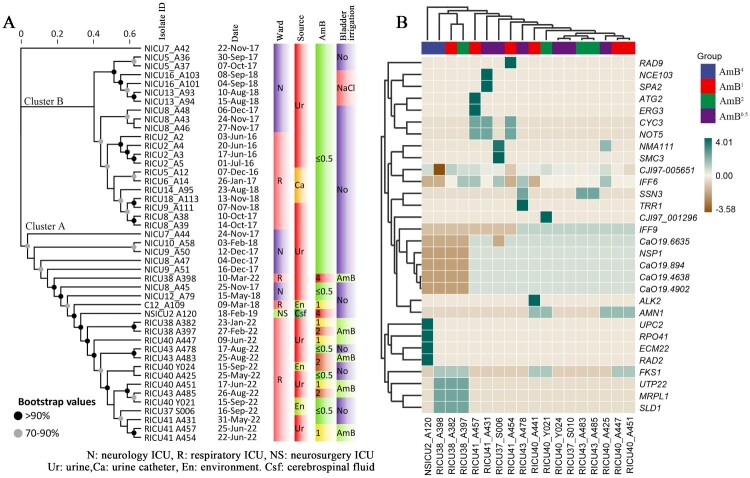
Genome sequencing of clinically isolated *C. auris*. (A) Molecular phylogenetic analysis of 45 isolates (including 16 serial isolates from all six patients in this study and 29 previously studied isolates). The phylogenetic tree constructed using 403 SNPs has two main clusters (Cluster A and Cluster B). (B) Heatmap of the 18 *C. auris* strains isolated in this study plotted against their 46 non-synonymously mutated genes using heatmap tools in the genescloud platform (https://www.genescloud.cn).

We also retrospectively reviewed 15 clinical cases prior to 2019. The six cases from the Neurosciences Intensive Care Unit (NICU) and the seven cases from the RICU were treated without bladder irrigation. To clear the urinary tract of *C. auris* infection or colonization, saline bladder irrigation was used in two cases from the NICU in 2018. There were no increases in the MIC values of AmB in patients treated with saline bladder irrigation compared to AmB bladder irrigation (Figure 2A). These results suggest a potential relationship between AmB bladder irrigation therapy and increased AmB MICs.

### Phylogenetic analyses of clinical C. auris isolates

Consistent with previous phylogenetic and population structure analyses, all isolates in this study belonged to the South African clade (clade III) [14] A total of 45 isolates (including 16 serial isolates from all six patients in this study and 29 previously studied isolates [14]) were phylogenetically analyzed to explore the origin of AmB^1^/AmB^2^ *C. auris* cases. The phylogenetic tree was constructed using 403 single nucleotide polymorphisms (SNPs) and revealed two main clusters (Cluster A and Cluster B). Cluster A consisted mainly of *C. auris* isolates involved in this study (AmB^1^ and AmB^2^ *C. auris*). Nine strains (9/15) were isolated from four RICU patients treated with AmB bladder irrigation. Isolates from each patient formed small independent branches rather than being grouped as resistant and sensitive strains. Surprisingly, we found that the most genetically similar isolates to the AmB^1^ and AmB^2^ *C. auris* strains were those isolated from NICU patients (e.g. NICU8_A45, NICU12_A79, among others) and those taken from the bedrail of RICU patients (C12_A109) before 2019. Cluster B was mainly composed of from previously isolated *C. auris* strains from RICU and NICU patients. All of the 29 *C. auris* clinical isolates in this branch showed sensitivity to AmB. The patients who were not treated with bladder irrigation therapy and those who used saline bladder irrigation were included in this branch (Figure 2A).

#### Genome-wide SNP locus analysis

A total of 77 SNP sites were found to have mutations, of which 46 were nonsynonymous and 31 were synonymous (Supplementary Table 2). Among them, one gene, *CJI97-005651,* possessed the most SNPs, with 7–11 nonsynonymous mutations (c.g3a.M1I, c.g4a.A2T, c.g47a.R16H, c.t50a.V17E, c.t53g.L18R, c.g56t.G19V, c.t61g.Y21D, c.t1247g.V416G, c.c1262g.P421R, c.c1265a.A422E, and c.g1276c.G426R) in all strains except strain A398, as well as five synonymous mutations. Four genes had 2–3 nonsynonymous mutations, *IFF6* (c.a146t.Y49F, c.g525c.M175I), *NMA111* (c.c940t.L314F, c.t2432a.L811H), *AMN1* (c.g845a.C282Y, stopgain c.c368a.S123X), and *FKS1* (c.t1915c.S639P, c.c1916t.S639F), while the other 26 genes had nonsynonymous mutations at a single SNP. Notably, certain nonsynonymous gene mutations may be associated with reduced susceptibility to AmB, such as those genes encoding delta-5-6 steroid desaturase *ERG3* (c.c923t.T308M), glycosylphosphatidylinositol (GPI)-anchored cell wall protein-encoding genes *IFF9* (c.t360a.F120L), *IFF6*, and RBR3 (c.g4154a.G1385D), sphingolipid delta-8 desaturase *SLD* (c.g1717c.V573L), DNA-damage repair-related genes *RAD2*(c.t2831c.L944P) and *RAD9* (c.a1964g.G655R), sterol uptake, and ergosterol biosynthesis genes *UPC2* (c.g95t.R32M). *RAD2* is a nucleotide excision repair (NER) gene, and NER mutants are very sensitive to UV-induced DNA damage [20]. The *RAD9* gene encodes a chromatin binding protein that acts as a signal transducer at DNA-damage checkpoints [21] and plays a role in DNA repair and metabolism. *UPC2* is a sterol uptake control protein and a transcription factor involved in the regulation of ergosterol biosynthesis and sterol uptake at the plasma membrane [22]. The tightly linked proteins encoded by 11 genes involved in *C. albicans* homologs in the protein interaction network (medium confidence = 0.4) are shown in Supplementary Figure 1A.

Nonsynonymous mutations in *RAD2, PRO41* (c.g121c.A41P), and *UPC2* were only found in AMB^2^ isolates (NSICU2_ A120). Strains with nonsynonymous mutations in *ERG3* were found only in the AmB^1^ group (RICU41_A457) and in *RAD9* in RICU41_A454 (Supplementary Figure 1B). Isolates RICU38_A382, RICU38_A397, and RICU38_A398 had mutations in *SLD1, UTP22* (c.c406t.L136F)*,* and *MRPL10* (c.t833c.V278A). These three strains also had a nonsynonymous mutation in *FKS1* (c.t1915c.S639P) as well as an echinocandin-resistant phenotype. As a result, these three mutations (*SLD1*, *UTP22,* and *MRPL10*) cannot be excluded from a potential association with echinocandin resistance in *C. auris* (Figure 2B) [23].

#### Transcriptomic analyses

**Figure 3.**
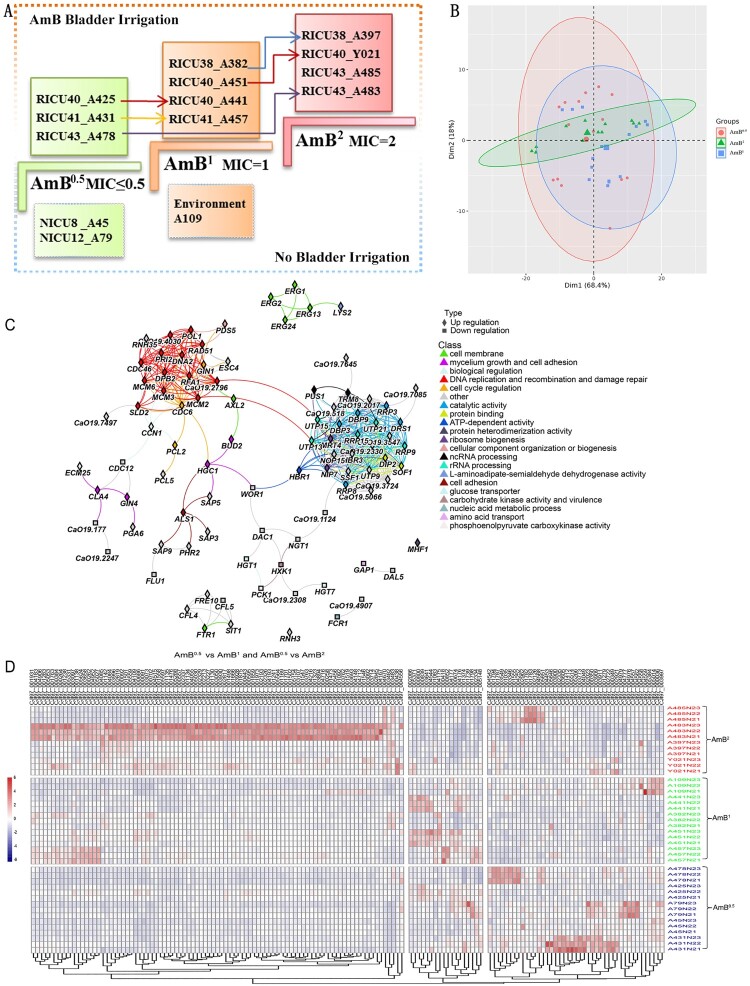
Transcriptomic data analysis. (A) Relationship between isolates and bladder irrigation. The 11 strains above were treated with AmB bladder irrigation and the three following strains were not treated with bladder irrigation. (B) Principal-component analysis (PCA) of normalized RNA-seq read counts from three biological replicates per isolate displays the level of correlation and the reproducibility among different biological replicates. R package DESeq2 software for PCA analysis, ellipse parameters set as follows: topN < - “500”; type < - “t”; c_level < -0.95; segments = 101; (C) Amino acid sequences of 152 identified DEGs were used in protein-protein interaction (PPI) network analysis. *Candida albicans* SC5314 homologous protein was used as a reference. PPI network was constructed using https://www.chiplot.online/normalTree.html. Grey circle: upregulated genes, grey forked cross: downregulated genes. Different colours represent different gene function classifications. (D) Heatmap of the 152 identified DEGs in AmB^0.5^, AmB^1^, and AmB^2^ isolates.

To further investigate the relationship between AmB bladder irrigation and reduced AmB sensitivity, 11 clinical isolates from four patients treated with AmB bladder irrigation were selected, and control isolates were selected from three previously studied *C. auris* isolates (NICU8_ A45, NICU12_ A79, and C12_ A109) for phylogenetic analyses (Figure 3A).

Multivariate principal- component analyses revealed similarities between biological replicates as well as differences among the *C. auris* isolates (Figure 3B). The biological replicates from each isolate clustered together, indicating a high level of data correlation, but the three groups did not cluster separately. There were 152 DEGs in the AmB^1^ and AmB^2^ *C. auris* isolates compared to the AmB^0.5^ isolates (Supplementary Table 3). From these 152 DEGs, the isolates were mapped according to the following groups: AmB^0.5^, AmB^1^, and AmB^2^. We found that 91 genes showed upregulated expression in the AmB^2^ group, 18 genes in the AmB^1^ group, and 43 genes in the AmB^0.5^ group (Figure 3D).

We also performed Gene Ontology and Kyoto Encyclopedia of Genes and Genomes (KEGG) enrichment analyses of the DEGs to gain insights into the overall differential transcriptional landscapes between the different isolates by RNA-seq (Supplementary Figure 2). KEGG analysis revealed that the DEGs were clustered in DNA replication-related pathways. Furthermore, we found that 13 genes (*CDC46, PRI2, DPB2, RAD51, MCM2, MCM3, MCM6, RFA1, DNA2, SLD2, POL1, CaO19.2796,* and *CaO19.4030)* were enriched in DNA-related pathways associated with DNA replication, DNA metabolism processes, DNA geometric changes, and DNA double-strand unwinding. Based on these annotations, a total of 31 genes were directly annotated for DNA replication by computational analysis. Nearly 9% of the DNA replication-related genes (13 out of 152 genes) were differentially expressed in AmB^1^ and AmB^2^ isolates compared to AmB^0.5^ isolates. Furthermore, nine genes related to integral membrane components, such as *ERG1, STL1, LAC12, HGT1, HGT7, GAP1, GAP2, FTR1,* and *RTA1*, were also differentially expressed. The cell adhesion-related genes*ALS4* and *ALS1* were also differentially expressed.

### DEG analysis of three groups (Total, RICU, RICU single case) between AmB^0.5^ vs. AmB^1^ and AmB^0.5^ vs. AmB^2^isolates

**Figure 4.**
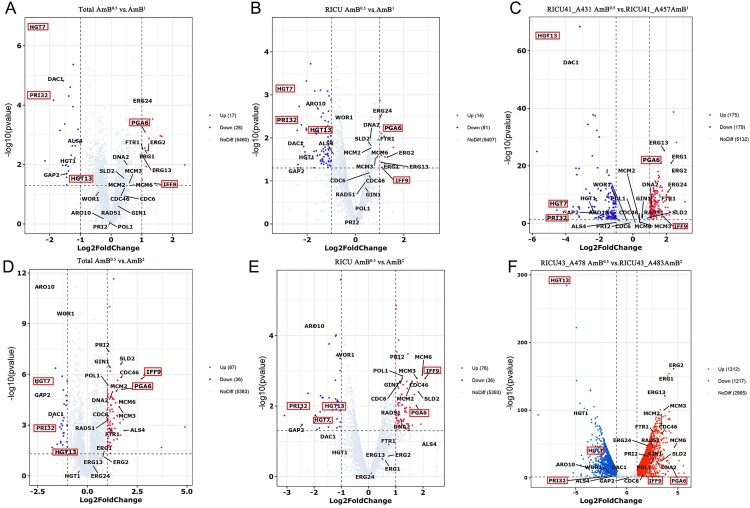
Transcriptomic profiling of three groups (total, RICU, RICU single case) between AmB^0.5^ vs. AmB^1^ and AmB^0.5^ vs. AmB^2^ isolates. Important genes are labelled and key genes are circled in red boxes. (A)Volcano map shows up- and downregulated genes in the total AmB^0.5^ group vs. total AmB^1^ group. Upregulated genes are indicated by red dots, downregulated genes are indicated by blue dots. (B) Volcano map shows up- and downregulated genes in the RICU AmB^0.5^ group vs. RICU AmB^1^ group. (C) Volcano map shows up- and downregulated genes in the RICU41_A431 AmB^0.5^ group vs. RICU41_A457 AmB^1^ group. (D) Volcano map shows up- and downregulated genes in total AmB^0.5^ group vs. total AmB^2^ group.(E) Volcano map shows up- and downregulated genes in the RICU AmB^0.5^ group vs. RICU AmB^2^ group.(F) Volcano map shows up- and downregulated genes in the RICU43_A478 AmB^0.5^ group vs. RICU43_A483AmB^2^ group.

**Figure 5.**
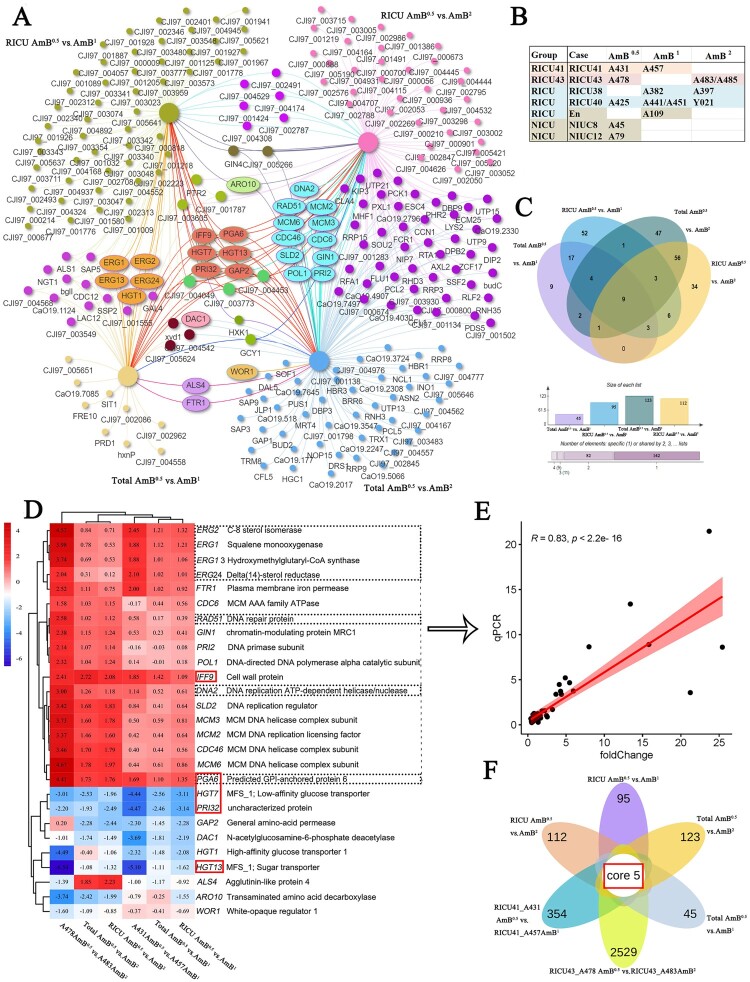
Comprehensive analysis of transcriptomic data. (A) Interactive venn network of four groups (total AmB^0.5^ vs. AmB^1^ group, RICU AmB^0.5^ vs. AmB^1^ group, total AmB^0.5^ vs. AmB^2^ group, and RICU AmB^0.5^ vs. AmB^2^ group) using venn diagram tools in the Evenn platform(http://ehbio.com/test/venn/). (B) Strain information for comprehensive analysis of transcriptome results. (C) Flower plot diagram of four groups (total AmB^0.5^ vs. AmB^1^ group, RICU AmB^0.5^ vs. AmB^1^ group, total AmB^0.5^ vs. AmB^2^ group, and RICU AmB^0.5^ vs. AmB^2^ group). (D) Heatmap of the six groups plotted against their DEGs using heatmap tools in the hiplot platform (https://hiplot.com.cn/home/index.html). (E) Correlation analysis of qPCR results and transcriptome data for seven genes including ERGs. (F) Flower plot diagram of six groups using venn diagram tools in the Evenn platform (http://ehbio.com/test/venn/). Important genes are labelled and key genes are circled in red boxes.

To explore the key factors of microevolution leading to reduced susceptibility to AmB in *C. auris*, we analyzed DEGs between the two groups (AmB^0.5^ vs. AmB^1^ and AmB^0.5^ vs. AmB^2^) at three levels: (1) comparison analysis of the total strains (RICU and NICU) (Figure 5B), where the Total AmB^0.5^ vs. AmB^1^ group had 17 upregulated genes and 28 downregulated genes, while the Total AmB^0.5^ vs. AmB^2^ group had 87 upregulated genes and 36 downregulated genes (Figure 4A, 4D); (2) comparison analysis of the RICU strains, and RICU AmB^0.5^ vs. AmB^1^ group had 14/81 upregulated/downregulated genes, while the RICU AmB^0.5^ vs. AmB^2^ group had 76/36 upregulated/downregulated genes (Figure 4B, 4E); and (3) Individual case from RICUs (RICU41_A431 vs. A457 and RICU43_A478 vs. A483) were analyzed separately, where 175/179 upregulated/downregulated genes; and 1312/1217 upregulated/ downregulated genes were found, respectively (Figure 4C, 4F). Finally, we performed comparative analyses between four groups (Figure 5A, 5C) and six groups (Figure 5F), identifying27 important genes (*ERG1*, etc.), which were related to cell membranes, cell adhesion, cell wall synthesis, DNA replication, recombination, and sugar transport (Figure 5D). Seven genes, including *ERG1*, were selected for qPCR validation, and their expression was consistent with transcriptome data (Figure 5E and Supplementary Table 4).

After two-by-two and four-group comparisons in the AmB^0.5^ vs. AmB^1^ groups, we identified important upregulated genes, including *ERG1, ERG2, ERG13,* and *ERG24*, which are involved in the ergosterol biosynthesis pathway. These genes were also associated with cell membranes (Figure 3C and 5D). In comparison, in the AmB^0.5^ vs. AmB^2^ group, important upregulated genes (i.e. *SLD2, PRI2, MCM2, MCM3, MCM6,* and *DNA2)* were related to DNA replication and recombination. These DNA-related genes are tightly linked in the network protein map (Figure 3C and 5D). *RAD51,* which is related to DNA repair, was also identified in the AmB^2^ group.

Most downregulated genes were implicated in glucose transporter proteins (*HGT1, HGT7,* and *HGT13*). *HGT1*, a multifunctional complement evasion molecule, also regulates hyphae formation, leading to the downregulation of complement activation. Genes such as *WOR1* and *HXK1* were differentially expressed, and although the protein interactions are poorly correlated, they are all associated with virulence. Enhanced resistance appears to be compensated for by the downregulated expression of virulence genes.

After the six-group overlap analysis, we identified five key genes: two upregulated genes *(IFF9,* and *PGA6)* and three downregulated genes (*HGT7, HGT13, and PRI32)* (Figure 5F)*.*

## Discussion

Since 2022, MIC values of clinical *C. auris* isolates against AmB have significantly increased. Among them, six AmB^1^ *C. auris* isolates (AmB MIC = 1 μg/mL) and six AmB^2^ *C. auris* isolates (AmB MIC = 2 μg/mL or 4 μg/mL) were identified, indicating a low level of drug resistance. Importantly, we also identified 11 *C. auris* strains with elevated MIC values for AmB from urine samples of four patients, all of whom had been treated with AmB bladder irrigation. Most AmB-susceptible cases of *C. auris* prior to 2019 had not been treated with saline bladder irrigation, with the exception of two patients in the NICU. We hypothesize that AmB resistance in *C. auris* may be associated with bladder irrigation. In addition, there are usually two regimens regarding the dosage of AmB bladder irrigation: 5 mg/L (following standard instructions) and 25–50 mg/L (recommended by guidelines [1]). Based on safety and other considerations, all cases in this study were treated with 5 mg/L AmB bladder irrigation, and this regimen is largely effective in the treatment of most *Candida*-induced urinary tract infections in the clinic [24]. Unfortunately, the results of this study showed that continuous bladder irrigation with 5 mg/L AmB for 24-hour may induce elevated AmB MIC in *C. auris*, thereby suggesting that high-dose AmB bladder irrigation (25–50 mg/L) should be used to eliminate *C. auris* colonization or infection in the absence of clinical contraindications.

Furthermore, we characterized the genetic evolution of AmB^1^ and AmB^2^ *C. auris* by genomic and transcriptomic analyses. First, we found that AmB^1^ and AmB^2^ *C. auris* isolates in this study developed mutations in *ERG3* (c.c923t.T308M) and *RAD2* (c.t2831c.L944P), which are associated with cell membranes and DNA damage repair, respectively. Whether these two nonsynonymous mutations are simply polymorphisms or whether they play an important role in AmB resistance requires further experimental confirmation. Secondly, we analyzed differences in transcript levels between AmB^1^ and AmB^2^ isolates compared to AmB^0.5^ isolates. We found that consistent with our genomic data, upregulated genes were related to two major categories: membrane lipids and DNA replication. Among them, the expression of membrane lipid-related genes (*ERG1, ERG2, ERG13,* and *ERG24*) was upregulated in the AmB^1^ group. AmB has been shown to bind or trap membrane ergosterol to trigger changes in membrane permeability by chelating ergosterol or by regulating channel function [25–27]. In contrast, qualitative and quantitative changes in membrane lipids and a reduction in total ergosterol content are closely associated with AmB resistance [28,29]. Additionally, DNA-related genes such as those involved in DNA replication (e.g. *DNA2*) and DNA metabolic processes (e.g. *PRI1*) were upregulated in AmB^2^ *C. auris* isolates. While the possibility that DNA replication may enhance AmB resistance in *Candida albicans* [30] has been suggested, the effect on reduced AmB sensitivity in *C. auris* has rarely been reported and needs further investigation. After the six-group overlap analysis, we identified five key genes: two upregulated genes (*IFF9* and *PGA6*) and three downregulated genes (*HGT7*, *HGT13*, and *PRI32*). The correlation between increased or decreased expression of these genes related to cell wall or membrane metabolism and AmB resistance deserves in-depth experimental studies to identify potential therapeutic targets.

Our phylogenetic analyses suggest that the overall evolution of *C. auris* isolates in China (Shenyang) occurred between 2016 and 2022, and that the *C. auris* isolates obtained before and after 2019 formed two major clusters (A and B). Cluster A included AmB^1^ and AmB^2^ isolates that were associated with individual evolution and were not derived from the same clone. Moreover, isolates obtained from the RICU were genetically more related to isolates from pre-2019 NICU patients. Therefore, the origin of strains with reduced AmB susceptibility in this study is complex and requires further investigation. Furthermore, one *C. auris* strain from the RICU setting (bedrail) was genetically related to these AmB^1^/ AmB^2^ strains. Another AmB^2^ strain was isolated from the groin of a patient (RICU40) in 2022, suggesting that AmB^1^ and AmB^2^ strains may be widely disseminated in healthcare settings, and the potential risk of an epidemic outbreak of AmB-resistant *C. auris* should not be underestimated.

We recognize several limitations to this study. First, the number of strains and cases involved was small. Therefore, further clarification is needed on whether low-dose AmB continuous bladder irrigation therapy is associated with increased resistance to AmB in *C. auris* by using a larger sample group and *in vitro* experimentation. Secondly, no *ERG6* mutation sites were identified in AmB^1^ and AmB^2^ *C. auris* isolates compared to AmB^0.5^. This may be related to the low level of resistance to AmB in the *C. auris* isolates in this study, pending further inclusion of high-level AmB-resistant isolates to explore the mechanisms of AmB resistance. Thirdly, this study used ATB Fungus 3 for testing drug sensitivity to AmB, which requires interpretation that may be subjective. Other widely used antifungal susceptibility tests, such as the SYO and automated Vitek 2 system (BioMerieux), greatly overestimate the resistance of *C. auris* to AmB [19,31]. Additionally, the true breakpoints or epidemiological cutoff values for AmB resistance in *C. auris* have not yet been established, which is an important clinical issue that needs urgent attention. Fourth, because the main target of AmB is ergosterol, *Candida* resistance to AmB should be accompanied by an absence of ergosterol in yeast cell membranes. In the present study, we were unable to detect sterol content due to experimental limitations. Therefore, gas chromatography-mass spectrometry (GC/MS) should be implemented in future studies to determine sterol content in isolate samples. Lastly, according to a recent study by Pezzotti G et al., different *C. auris* subclades have unique ergosterol/ergostane fractions analyzed by Raman spectroscopy, which significantly impact AmB resistance [32–34]. The *C. auris* clusters in this study all belonged to Clade III and had different MICs for AmB. Future efforts should focus on studying the Raman spectroscopy characteristics of *C. auris* with reduced AmB susceptibility, providing a powerful tool for the rapid and accurate detection of AmB-resistant strains of *C. auris*.

In conclusion, we have revealed important insights into the microevolution of reduced AmB sensitivity in *C. auris* cases treated with continuous AmB bladder irrigation in China (Shenyang). This suggests that clinicians should closely monitor *C. auris* isolates from urine specimens for changes in reduced AmB sensitivity or resistance, especially when using low-dose AmB bladder irrigation regimens. These findings will inform strategies for the elimination of *C. auris* and the prevention of further emergence of AmB resistance, which remains a challenging task.

## Supplementary Material

Supplementary Table1.docx

supplementary data1 Basic expression value.xlsx

CWS_Editorial_Certificate.pdf

Supplementary table 3.xlsx

Supplementary table 4 Correlation analysis between qPCR and transcriptome.xlsx

Supplementary Figure1.docx

Supplementary table 2.xlsx

## Data Availability

The raw sequence data reported in this work have been deposited in the Genome Sequence Archive (Genomics, Proteomics & Bioinformatics 2021) in National Genomics Data Center (Nucleic Acids Res 2022), the China National Center for Bioinformation/Beijing Institute of Genomics, as well as the Chinese Academy of Sciences (GSA: CRA015722, CRA015704, CRA015934, and CRA016185). All data are publicly accessible at https://ngdc.cncb.ac.cn/gsa.

## References

[1] Pappas PG, Kauffman CA, Andes DR, et al. Clinical practice guideline for the management of candidiasis: 2016 update by the Infectious Diseases Society of America. Clin Infect Dis. 2016;62(4):e1–e50. doi:10.1093/cid/civ933 26679628 PMC4725385

[2] Vincent BM, Lancaster AK, Scherz-Shouval R, et al. Fitness trade-offs restrict the evolution of resistance to amphotericin B. PLOS Biol. 2013;11(10):e1001692. doi:10.1371/journal.pbio.1001692 24204207 PMC3812114

[3] Ahmad S, Joseph L, Parker JE, et al. ERG6 and ERG2 are major targets conferring reduced susceptibility to amphotericin B in clinical *Candida glabrata* Isolates in Kuwait. Antimicrob Agents Chemother. 2019;63(2):e01900–18. doi:10.1128/AAC.01900-18 30455247 PMC6355561

[4] Chen J, Tian S, Han X, et al. Is the superbug fungus really so scary? A systematic review and meta-analysis of global epidemiology and mortality of *Candida auris*. BMC Infect Dis. 2020;20(1):827. doi:10.1186/s12879-020-05543-0 33176724 PMC7656719

[5] Asadzadeh M, Alfouzan W, Parker JE, et al. Molecular characterization and sterol profiles identify nonsynonymous mutations in *ERG2* as a major mechanism conferring reduced susceptibility to amphotericin B in *Candida kefyr*. Microbiol Spectr. 2023;11(4):e0147423. doi:10.1128/spectrum.01474-23 37358415 PMC10434000

[6] Kannan A, Asner SA, Trachsel E, et al. Comparative genomics for the elucidation of multidrug resistance in *Candida lusitaniae*. mBio. 2019;10(6):e02512–19. doi:10.1128/mBio.02512-19 31874914 PMC6935856

[7] Vandeputte P, Tronchin G, Larcher G, et al. A nonsense mutation in the *ERG6* gene leads to reduced susceptibility to polyenes in a clinical isolate of *Candida glabrata*. Antimicrob Agents Chemother. 2008;52(10):3701–3709. doi:10.1128/AAC.00423-08 18694952 PMC2565872

[8] Hull CM, Parker JE, Bader O, et al. Facultative sterol uptake in an ergosterol-deficient clinical isolate of *Candida glabrata* harboring a missense mutation in *ERG11* and exhibiting cross-resistance to azoles and amphotericin B. Antimicrob Agents Chemother. 2012;56(8):4223–4232. doi:10.1128/AAC.06253-11 22615281 PMC3421581

[9] Hull CM, Bader O, Parker JE, et al. Two clinical isolates of *Candida glabrata* exhibiting reduced sensitivity to amphotericin B both harbor mutations in ERG2. Antimicrob Agents Chemother. 2012;56(12):6417–6421. doi:10.1128/AAC.01145-12 23027188 PMC3497184

[10] Ben Abid F, Salah H, Sundararaju S, et al. Molecular characterization of *Candida auris* outbreak isolates in Qatar from patients with COVID-19 reveals the emergence of isolates resistant to three classes of antifungal drugs. Clin Microbiol Infect. 2023 Aug;29(8):1083.e1–1083.e7. doi:10.1016/j.cmi.2023.04.025 PMC10132836 37116861

[11] Carolus H, Pierson S, Muñoz JF, et al. Genome-wide analysis of experimentally evolved *Candida auris* reveals multiple novel mechanisms of multidrug resistance. mBio. 2021 Apr 5;12(2):e03333–20. doi:10.1128/mBio.03333-20 33820824 PMC8092288

[12] Rybak JM, Barker KS, Muñoz JF, et al. In vivo emergence of high-level resistance during treatment reveals the first identified mechanism of amphotericin B resistance in *Candida auris*. Clin Microbiol Infect. 2022;28(6):838–843. doi:10.1016/j.cmi.2021.11.024 34915074 PMC9467277

[13] Shivarathri R, Jenull S, Chauhan M, et al. Comparative transcriptomics reveal possible mechanisms of amphotericin B resistance in *Candida auris*. Antimicrob Agents Chemother. 2022;66(6):e0227621. doi:10.1128/aac.02276-21 35652307 PMC9211394

[14] Tian S, Bing J, Chu Y, et al. Genomic epidemiology of *Candida auris* in a general hospital in Shenyang, China: a three-year surveillance study. Emerg Microbes Infect. 2021;10(1):1088–1096. doi:10.1080/22221751.2021.1934557 34027824 PMC8183536

[15] Chen Y, Zhao J, Han L, et al. Emergency of fungemia cases caused by fluconazole-resistant *Candida auris* in Beijing, China. J Infect. 2018;77(6):561–571. doi:10.1016/j.jinf.2018.09.002 30219662

[16] Wang K, Li M, Hakonarson H. ANNOVAR: functional annotation of genetic variants from high-throughput sequencing data. Nucleic Acids Res. 2010;38(16):e164. doi:10.1093/nar/gkq603 20601685 PMC2938201

[17] Tamura K, Nei M. Estimation of the number of nucleotide substitutions in the control region of mitochondrial DNA in humans and chimpanzees. Mol Biol Evol. 1993;10(3):512–526. doi:10.1093/oxfordjournals.molbev.a040023 8336541

[18] Kumar S, Stecher G, Mega TK. *Molecular Evolutionary Genetics Analysis*. version 7.0 for Bigger Datasets. Mol Biol Evol. 2016;33(7):1870–1874. doi:10.1093/molbev/msw054 27004904 PMC8210823

[19] Siopi M, Peroukidou I, Beredaki M-I, et al. Overestimation of amphotericin B resistance in *Candida auris* with Sensititre YeastOne antifungal susceptibility testing: a need for adjustment for correct interpretation. Microbiol Spectr. 2023;11(3):e0443122. doi:10.1128/spectrum.04431-22 37036351 PMC10269614

[20] Legrand M, Chan CL, Jauert PA, et al. Analysis of base excision and nucleotide excision repair in *Candida albicans*. Microbiology (Reading). 2008;154(8):2446–2456. doi:10.1099/mic.0.2008/017616-0 18667577

[21] Shi Q-M, Wang Y-M, Zheng X-D, et al. Critical role of DNA checkpoints in mediating genotoxic-stress-induced filamentous growth in *Candida albicans*. Mol Biol Cell. 2007;18(3):815–826. doi:10.1091/mbc.e06-05-0442 17182857 PMC1805102

[22] Li J, Aubry L, Brandalise D, et al. Upc2-mediated mechanisms of azole resistance in *Candida auris*. Microbiol Spectr. 2024;12(2):e0352623. doi:10.1128/spectrum.03526-23 38206035 PMC10845950

[23] Tian S, Wu Y, Li H, et al. Evolutionary accumulation of *FKS1* mutations from clinical echinocandin-resistant *Candida auris*. Emerg Microbes Infect. 2024 Dec;13(1):2377584. doi:10.1080/22221751.2024.2377584. Epub 2024 Jul 22. 38989545 PMC11265302

[24] Griffith N, Danziger L. *Candida auris* urinary tract infections and possible treatment. Antibiotics (Basel). 2020;9(12):898. doi:10.3390/antibiotics9120898 33322761 PMC7764735

[25] Anderson TM, Clay MC, Cioffi AG, et al. Amphotericin forms an extramembranous and fungicidal sterol sponge. Nat Chem Biol. 2014;10(5):400–406. doi:10.1038/nchembio.1496 24681535 PMC3992202

[26] Guo X, Zhang J, Li X, et al. Sterol sponge mechanism is conserved for glycosylated polyene macrolides. ACS Cent Sci. 2021;7(5):781–791. doi:10.1021/acscentsci.1c00148 34079896 PMC8161476

[27] Muñoz JF, Gade L, Chow NA, et al. Cuomo CA.Genomic insights into multidrug-resistance, mating and virulence in *Candida auris* and related emerging species. Nat Commun. 2018 Dec 17;9(1):5346. doi:10.1038/s41467-018-07779-6 30559369 PMC6297351

[28] Geber A, Hitchcock CA, Swartz JE, et al. Deletion of the *Candida glabrata ERG3* and *ERG11* genes: effect on cell viability, cell growth, sterol composition, and antifungal susceptibility. Antimicrob Agents Chemother. 1995;39(12):2708–2717. doi:10.1128/AAC.39.12.2708 8593007 PMC163017

[29] Sanglard D, Ischer F, Parkinson T, et al. *Candida albicans* mutations in the ergosterol biosynthetic pathway and resistance to several antifungal agents. Antimicrob Agents Chemother. 2003;47(8):2404–2412. doi:10.1128/AAC.47.8.2404-2412.2003 12878497 PMC166068

[30] Uppuluri P, Chaffin WL. Defining *Candida albicans* stationary phase by cellular and DNA replication, gene expression and regulation. Mol Microbiol. 2007;64(6):1572–1586. doi:10.1111/j.1365-2958.2007.05760.x 17555439

[31] Siopi M, Pachoulis I, Leventaki S, et al. Evaluation of the Vitek 2 system for antifungal susceptibility testing of *Candida auris* using a representative international panel of clinical isolates: overestimation of amphotericin B resistance and underestimation of fluconazole resistance. J Clin Microbiol. 2024;62(4):e0152823. doi:10.1128/jcm.01528-23 38501836 PMC11005389

[32] Pezzotti G, Kobara M, Asai T, et al. Raman Imaging of Pathogenic *Candida auris*: Visualization of Structural Characteristics and Machine-Learning Identification. Front Microbiol. 2021 Nov 12;12:769597. doi:10.3389/fmicb.2021.769597. eCollection 2021. 34867902 PMC8633489

[33] Pezzotti G, Kobara M, Nakaya T, et al. Raman study of pathogenic *Candida auris*: imaging metabolic machineries in reaction to antifungal drugs. Front Microbiol. 2022;25(13):896359. doi:10.3389/fmicb.2022.896359 PMC9175029 35694304

[34] Pezzotti G, Kobara M, Nakaya T, et al. Raman metabolomics of *Candida auris* clades: profiling and barcode identification. Int J Mol Sci. 2022;23(19):11736. doi:10.3390/ijms231911736 36233043 PMC9569935

