# Supplementary material for: Genetic microevolution of clinical Candida auris with reduced Amphotericin B sensitivity in China
Source: Emerg Microbes Infect. 2024 Sep 5;13(1):2398596. doi: 10.1080/22221751.2024.2398596 (PMC11385638; doi:10.1080/22221751.2024.2398596)
Supplement: Supplementary Table1.docx [file TEMI_A_2398596_SM9541.docx]

**Supplementary Table 1: Clinical information and drug sensitivity results in *C.auris* isolates (n=18)**

| Patient  (Sex/Age) | Isolate | Ward | Source | MIC (μg/mL) (YeastOne) | | | | | | | | | MIC(μg/mL)  (Fungus3) |
| --- | --- | --- | --- | --- | --- | --- | --- | --- | --- | --- | --- | --- | --- |
|  |  |  |  | FLU | ITC | POS | VOR | 5-FG | MFG | CFG | AFG | AMB | AMB |
| NSICU2  (F/65) | A120 | NSICU | csf | >256 | 0.5 | 0.25 | 4 | 0.12 | 0.12 | 0.12 | 0.25 | 8 | 4 |
| RICU38  (M/54) | A382 | RICU | ur | 256 | 0.06 | 0.06 | 0.5 | 0.12 | 8 | 8 | 4 | 2 | 1 |
|  | A397 | RICU | ur | >256 | 0.12 | 0.06 | 1 | 4 | 4 | 2 | 1 | 4 | 2 |
|  | A398 | RICU | ur | >256 | 0.12 | 0.06 | 1 | 4 | 4 | 2 | 2 | 8 | 4 |
| RICU40  (M/70) | A425 | RICU | ur | >256 | 0.25 | 0.06 | 1 | 0.12 | 0.12 | 0.25 | 0.25 | 1 | ≤0.5 |
|  | A441 | RICU | ur | >256 | 0.25 | 0.06 | 1 | 0.12 | 8 | 2 | 8 | 2 | 1 |
|  | A447 | RICU | ur | >256 | 0.25 | 0.06 | 1 | 0.25 | 8 | 2 | 8 | 2 | 1 |
|  | A451 | RICU | ur | >256 | 0.25 | 0.06 | 1 | 0.25 | 8 | 2 | 8 | 2 | 1 |
|  | Y 0021 | RICU | en^a^ | 256 | 0.25 | 0.06 | 0.5 | 0.25 | 8 | 8 | 4 | 4 | 2 |
|  | Y 0024 | RICU | en^b^ | 256 | 0.25 | 0.06 | 0.5 | 0.12 | 0.12 | 0.12 | 0.25 | 1 | ≤0.5 |
| RICU41  (F/50) | A431 | RICU | ur | >256 | 16 | 8 | 8 | 0.25 | 0.25 | 0.25 | 0.25 | 1 | ≤0.5 |
|  | A454 | RICU | ur | >256 | 8 | 4 | 8 | 0.12 | 0.25 | 0.25 | 0.25 | 2 | 1 |
|  | A457 | RICU | ur | >256 | 16 | 8 | 8 | 0.25 | 0.25 | 0.5 | 0.25 | 2 | 1 |
| RICU43  (F/78) | A478 | RICU | ur | >256 | 0.25 | 0.12 | 2 | 0.12 | 0.06 | 0.06 | 0.12 | 1 | ≤0.5 |
|  | A483 | RICU | ur | >256 | 0.25 | 0.12 | 4 | 0.12 | 0.12 | 0.12 | 0.12 | 4 | 2 |
|  | A485 | RICU | ur | >256 | 0.25 | 0.12 | 2 | 0.12 | 0.25 | 0.25 | 0.25 | 4 | 2 |
| RICU37  (M/91) | Y 0010 | RICU | en^a^ | 128 | 0.25 | 0.06 | 0.5 | 0.25 | 0.12 | 0.12 | 0.25 | 1 | ≤0.5 |
|  | Y 0006 | RICU | en^c^ | 128 | 0.12 | 0.06 | 0.5 | 0.25 | 0.12 | 0.12 | 0.12 | 1 | ≤0.5 |
| - | ATCC6258 | - | - | 32 | 0.5 | 0.25 | 0.25 | 8 | 0.25 | 0.5 | 0.12 | 1 | ≤0.5 |
| - | ATCC22019 | - | - | 2 | 0.25 | 0.06 | 0.03 | 0.25 | 1 | 0.5 | 0.5 | 1 | ≤0.5 |

Note: SICU Surgical ICU; RICU Respiratory ICU; csf cerebrospinal fluid; en environment; ur urine; a groin；b axilla; c bed gear.

The quality control range for AmB: 0.5 to 2 μg/mL for ATCC 6258 ***Candida krusei*** and 0.25 to 2 μg/mL for ATCC 22019 ***Candida parapsilosis*** (Sensititre YeastOne).

The quality control range for AmB: ≤0.5-2 μg/mL for ATCC 6258 ***Candida krusei*** and ≤0.5 μg/mL for ATCC 22019 ***Candida parapsilosis*** (ATB Fungus3).
