# Supplementary material for: Genetic microevolution of clinical Candida auris with reduced Amphotericin B sensitivity in China
Source: Emerg Microbes Infect. 2024 Sep 5;13(1):2398596. doi: 10.1080/22221751.2024.2398596 (PMC11385638; doi:10.1080/22221751.2024.2398596)
Supplement: CWS_Editorial_Certificate.pdf [file TEMI_A_2398596_SM9539.pdf]

EDITORIAL CERTIFICATE

This document certifies that the manuscript below was edited for correct English language usage, grammar, punctuation and spelling by qualified native English speaking editors at Charlesworth Author Services.

Paper Title:

AmB resistance and genetic microevolution of clinical Candida auris in China

Author:

sufei tian

Date certificate issued:

July 25, 2024

cwauthors.com
