# Supplementary material for: Genetic microevolution of clinical Candida auris with reduced Amphotericin B sensitivity in China
Source: Emerg Microbes Infect. 2024 Sep 5;13(1):2398596. doi: 10.1080/22221751.2024.2398596 (PMC11385638; doi:10.1080/22221751.2024.2398596)
Supplement: Supplementary Figure1.docx [file TEMI_A_2398596_SM9536.docx]

**Supplementary Figure 1**

**
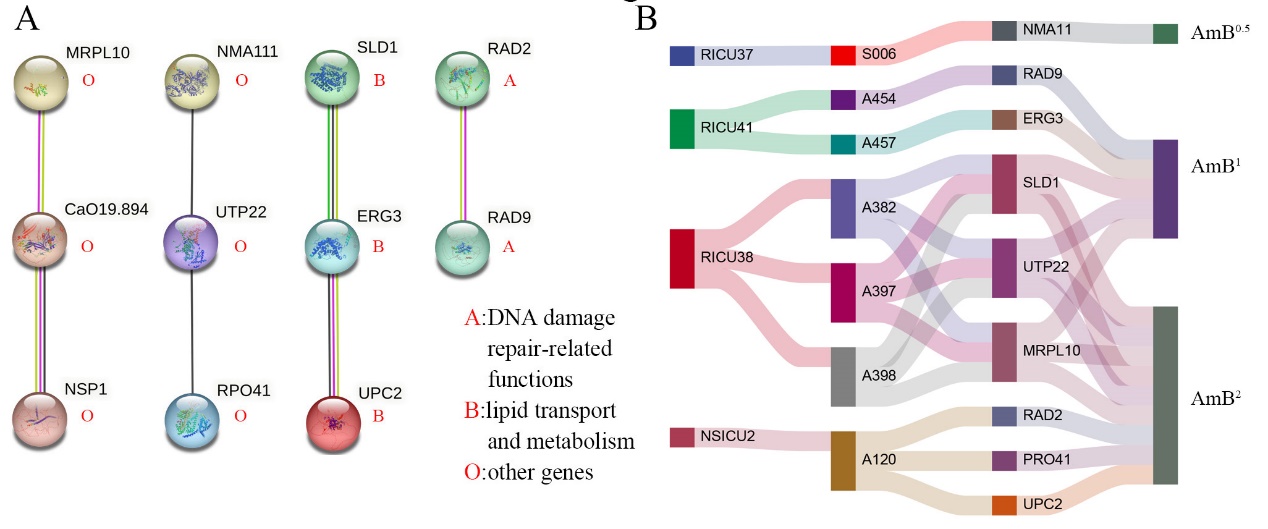
**

**(A)** The protein network interaction map involves non-synonymous mutant genes. There are 11 tightly linked proteins encoded by genes involved in *C. albicans* homologs in the protein interaction network (medium confidence = 0.4). These 11 genes were split into three categories including A, B and C. **(B)** The relationship between non-synonymous mutations and AmB drug resistance between different patient isolates.

**Supplementary Figure 2A** Functional annotation of 152 DEGs using KEGG.


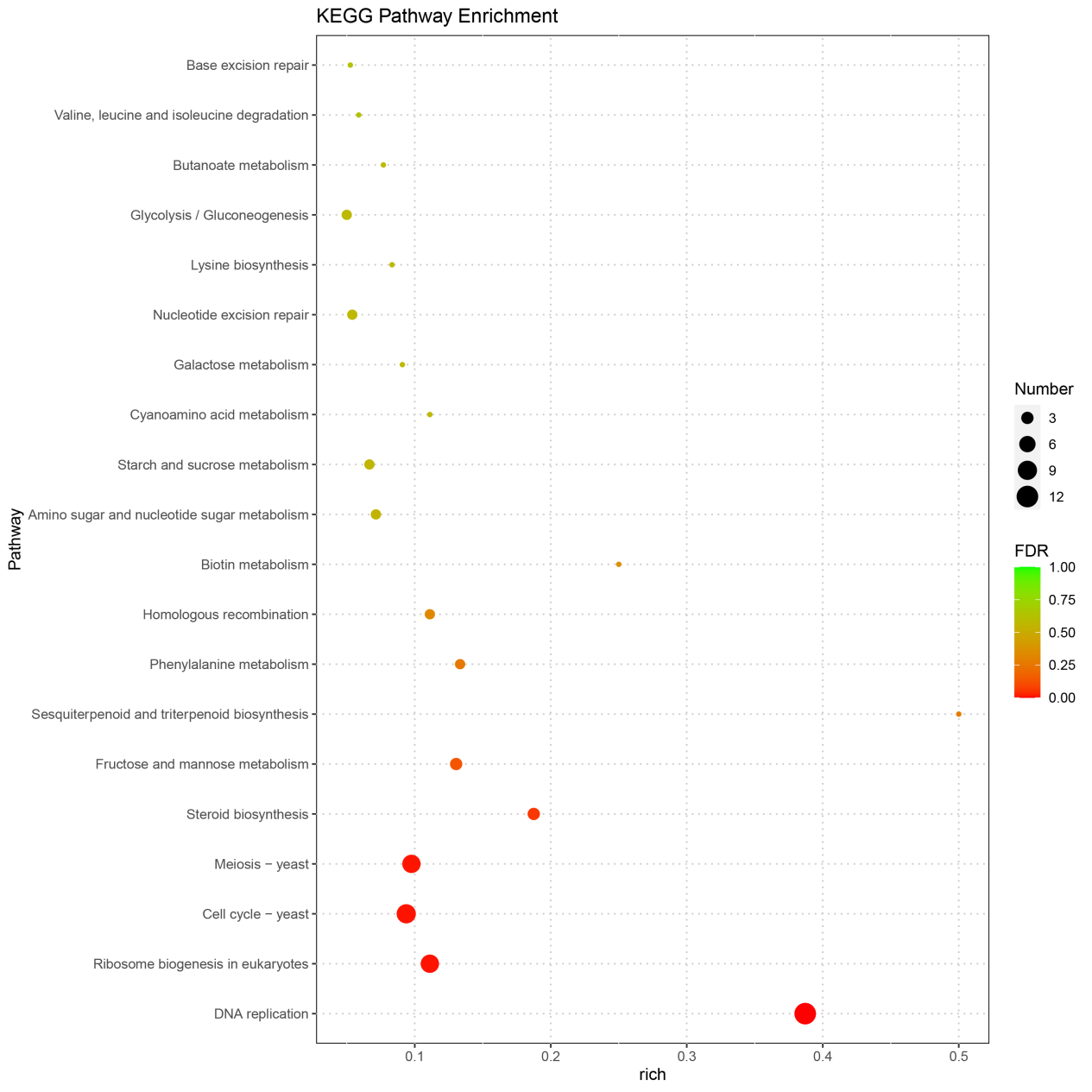


**Supplementary Figure 2A** Functional annotation of 152 DEGs using KEGG. The KEGG functional classification revealed that the predicted DEGs were associated with DNA replication, ribosome biogenesis in eukaryotes, cell cycle, meiosis, and steroid biosynthesis.

**Supplementary Figure 2B** Functional annotation of 152 DEGs using the GO database.


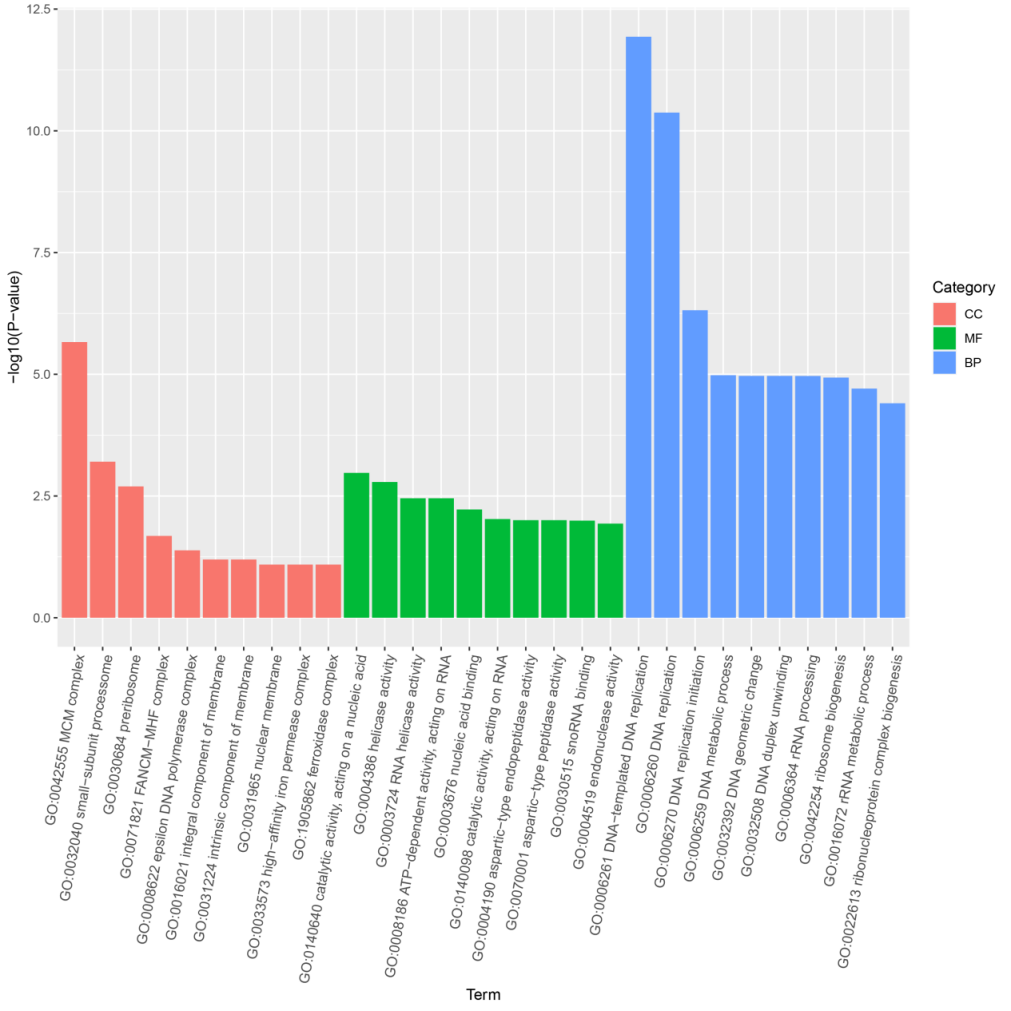


**Supplementary Figure 2B** Functional annotation of 152 DEGs using the GO database. We found a significant enrichment of GO biological process in all AmB^E^ and AmB^R^ strains compared to the AmB^S^ isolates, including cellular component, biological process, and molecular function. The DNA related genes (e.g., DNA replication and DNA metabolic process) were enriched terms associated with “biological processes”. The membrane related genes (e.g., integral component and intrinsic component of membrane) were enriched terms associated with “cellular component”.

**Supplementary 3 qPCR experimental procedure**

- 1. **RNA quality detection**

A complete RNA electrophoretic map generally has 3 bands, which are 28S, 18S and 5SrRNA from top to bottom. The purpose of electrophoresis is to detect the integrity of 28S and 18S. If all 3 bands are present and the main band is clear, single and bright, the RNA is considered to be good.

**3.2 Synthesis of first strand of cDNA**

The qualified and quantified total RNA was reverse-transcribed into cDNA (PrimeScript TM 1st stand cDNA Synthesis Kit) and the following reaction mixture was added to the test tube in the ice bath：

**Supplementary Table 3-1 The retro transcriptional response system 1**

| **reagent** | **system** |
| --- | --- |
| Template RNA：total RNA | 1 μg |
| primer：Oligo(dT) (50 uM) | 1 μl |
| dNTP Mix(10 mmol/L) | 1 μl |

Add RNase free dH2O to 10 μl, mix and incubate at 65 ℃ for 5 min, then quickly ice bath.

Add the following reaction mixture to the tube of the ice bath：

**Supplementary Table3-2 The retro transcriptional response system 2**

| **Reagent** | **System** |
| --- | --- |
| Template RNA Primer Mixture | 10μl |
| 5×Reaction Buffer | 4 μl |
| RNase Inhibitor (40 U/μl) | 0.5 μl |
| MMLV RT（200 U/μl） | 1 μl |
| RNase free dH_2_O | Up to 20 μl |

Reaction mixture at 42 ℃ for 30-60 min

The reaction was heated at 95℃ for 5min and then placed on ice for subsequent experiments or cryopreservation.

- 1. **Fluorescence quantitative PCR reaction**

**Supplementary Table3-3 primers used in the experiment**

| **Primer id** | **Primer sequence (5'to3')** |
| --- | --- |
| *PRI2*-F | GAGAACCTTCATATCAACCA |
| *PRI2*-R | TAGACACAGCCTTCGTAT |
| *DNA2*-F | GTGATAGTAAGAGAAGAAG |
| *DNA2*-R | ATTAGATAAGCGATAACG |
| *MCM6*-F | TGTGATATGTGCTCTACT |
| *MCM6*-R | GTTCAATGTCCAATACGA |
| *RAD51*-F | TAGAGGAGAGCAGAGAAT |
| *RAD51*-R | TCATAGATGGCAAACACA |
| *PGA6*-F | TCCGAGAGCGATGTCTAC |
| *PGA6*-R | TCCTGAGCGTAAGAAGTGT |
| *ERG1*-F | GTGGACGGCTACTATATC |
| *ERG1*-R | GTTGGTCTTGTCACATTC |
| *ERG2*-F | ACTTCTGTGGGTACTGAG |
| *ERG2*-R | ATTCCTTGCCGTATAAGATG |
| *ERG13*-F | GTATGTTGTATAACGACTTC |
| *ERG13*-R | TATGAGATGTTGGAGACT |
| *ERG24*-F | ATCTTCATCACCATCATC |
| *ERG24*-R | GTTACCAGAGTTACCATTA |
| actin（nei）-F | ATTCTGGAGATGGTGTTA |
| actin（nei）-R | TCAAGTAGTCAGTCAAGT |

PCR reaction system was configured.

For each target gene and steward gene, the cDNA template of the sample was selected for PCR reaction：

**Supplementary Table3-4: qPCR computer reaction system**

| **Reagent** | **System** |
| --- | --- |
| 2×SYBR real-time PCR premixture | 10μl |
| 10uM 的PCR primer F | 0.4 µl |
| 10uM 的PCR primer R | 0.4 µl |
| cDNA | 1 μl |
| RNase free dH_2_O | Up to 20 μl |

- 1. **Real-time PCR reaction**

The PCR reaction solution configured according to reaction system A was placed on the RealtimePCR instrument for PCR reaction, reaction procedure：

95℃ 5 min

95℃ 15 sec

40 cycles

60℃ 30 sec
